# Low Serum Testosterone Levels Are Associated with Elevated Urinary Mandelic Acid, and Strontium Levels in Adult Men According to the US 2011–2012 National Health and Nutrition Examination Survey

**DOI:** 10.1371/journal.pone.0127451

**Published:** 2015-05-21

**Authors:** Cheng Xu, Qian Liu, Hui Liu, Paul Héroux, Qunwei Zhang, Zhao-Yan Jiang, Aihua Gu

**Affiliations:** 1 State Key Laboratory of Reproductive Medicine, Institute of Toxicology, Nanjing Medical University, Nanjing, China; 2 Key Laboratory of Modern Toxicology of Ministry of Education, School of Public Health, Nanjing Medical University, Nanjing, China; 3 InVitroPlus Laboratory, Department of Surgery, Royal Victoria Hospital, Montreal, QC, Canada; 4 Department of Epidemiology, Biostatistics and Occupational Health, McGill University, Montreal, QC, Canada; 5 Department of Environmental and Occupational Health Sciences, School of Public Health and Information Health Sciences, University of Louisville, Louisville, KY, 40292, United States of America; 6 Department of Surgery, Ruijin Hospital, Shanghai JiaoTong University School of Medicine, Shanghai, China; Gentofte University Hospital, DENMARK

## Abstract

**Background:**

Little is known regarding the effects of environmental exposure of chemicals on androgenic system in the general population. We studied 5,107 subjects included in the National Health and Nutrition Examination Survey (2011–2012).

**Methods:**

Urinary, serum, and blood levels of 15 subclasses comprising 110 individual chemicals were analyzed for their association with serum testosterone levels. The subjects were divided into high and low testosterone groups according to the median testosterone concentration (374.51 ng/dL). Odds ratios (ORs) of individual chemicals in association with testosterone were estimated using logistic regression after adjusting for age, ethnicity, cotinine, body mass index, creatinine, alcohol, and the poverty income ratio.

**Results:**

Adjusted ORs for the highest versus lowest quartiles of exposure were 2.12 (95% CI: 1.07, 4.21; *P_trend_* = 0.044), 1.84 (95% CI: 1.02, 3.34; *P_trend_* = 0.018) for the association between urinary mandelic acid, and strontium quartiles with low testosterone concentrations in adult men, respectively. However, no association was observed for the remaining chemicals with testosterone.

**Conclusions:**

The National Health and Nutrition Examination Survey data suggest that elevations in urinary mandelic acid, and strontium levels are negatively related to low serum testosterone levels in adult men.

## Introduction

Testosterone, an androgen steroid, plays a crucial role in the growth of male reproductive tissues and other secondary sexual characteristics. Essential functions of testosterone include differentiation of spermatogonia, regulation of the hypothalamic–pituitary–adrenal axis response, maintenance of muscle trophism, cognitive competence, and physical strength.

Changes in testosterone levels in men may lead to several pathological states. Low testosterone levels are associated with defects in the cardiovascular system [1] and metabolic syndrome, including a risk of diabetes and osteoporosis [2, 3]. Male infertility (oligospermia or azoospermia) has also been linked with low testosterone levels. However, testosterone replacement therapy may increase the risk of myocardial infarction and ischemic stroke [4, 5]. Therefore, maintaining testosterone levels at physiological levels is of major importance.

In adult men, the normal level of testosterone ranges from 300 to 1,000 ng/dL [6]. Testosterone can be affected by some exogenous compounds, by interfering with several biological processes of testosterone [7, 8]. Most of the evidence on effects of exogenous compounds were obtained by rodent studies at high doses that elicited certain toxic endpoints, such as organ damage [9, 10]. These studies were sometimes augmented by epidemiological observations that associated environmental exposure with certain health endpoints. Exposure to low doses of chemicals and physical agents may threaten human health. Therefore, more attention is required to obtain an environmental chemical profile and evaluate their potential side effects.

Recent data from human population studies have shown that some environmental chemicals, even at low doses, disturb reproductive hormone levels, including testosterone concentrations [11–15]. Elevated urinary manganese and zinc levels, as well as trihalomethane exposure, can reduce serum testosterone concentrations [11, 16]. Perfluorooctane sulphonate (PFOS) levels might also be associated with circulatory testosterone levels in adult men [12]. A Danish population study showed that PFOS in serum is negatively associated with testosterone levels in healthy men (n = 105), whereas the same study showed non-significant trends regarding altered testosterone levels with high PFOS levels in men (n = 247) [15]. The inconsistency between these studies is difficult to explain, but might be due to a limited sample size or the presence of unadjusted confounders. If various environmental chemicals are taken into consideration, this makes the situation more complicated. To date, over 300 such chemicals and their metabolites have been detected in human samples (e.g., urine, blood, serum, breast milk, and meconium). Whether these chemicals are associated with testosterone levels has not been fully clarified yet. How to best analyze epidemiological data on combined exposure remains a large challenge. The major intention of the National Health and Nutrition Examination Survey (NHANES) is to monitor trends in environmental exposure and risky behavior.

To find out suggestive evidences of potential reproductive damage effect by environmental chemicals at epidemiological level, we comprehensively investigated the association between all detectable urinary or blood chemicals and serum testosterone levels using the NHANES data from 2011–2012.

## Materials and Methods

### Study design and participants

NHANES is conducted by the US Centers for Disease Control and Prevention, and is designed to assess health and nutrition in a nationally representative sample of the civilian, non-institutionalized US population. From 2011–2012, 13,431 people were selected for NHANES in 30 different study locations [17, 18].

**Fig 1:**
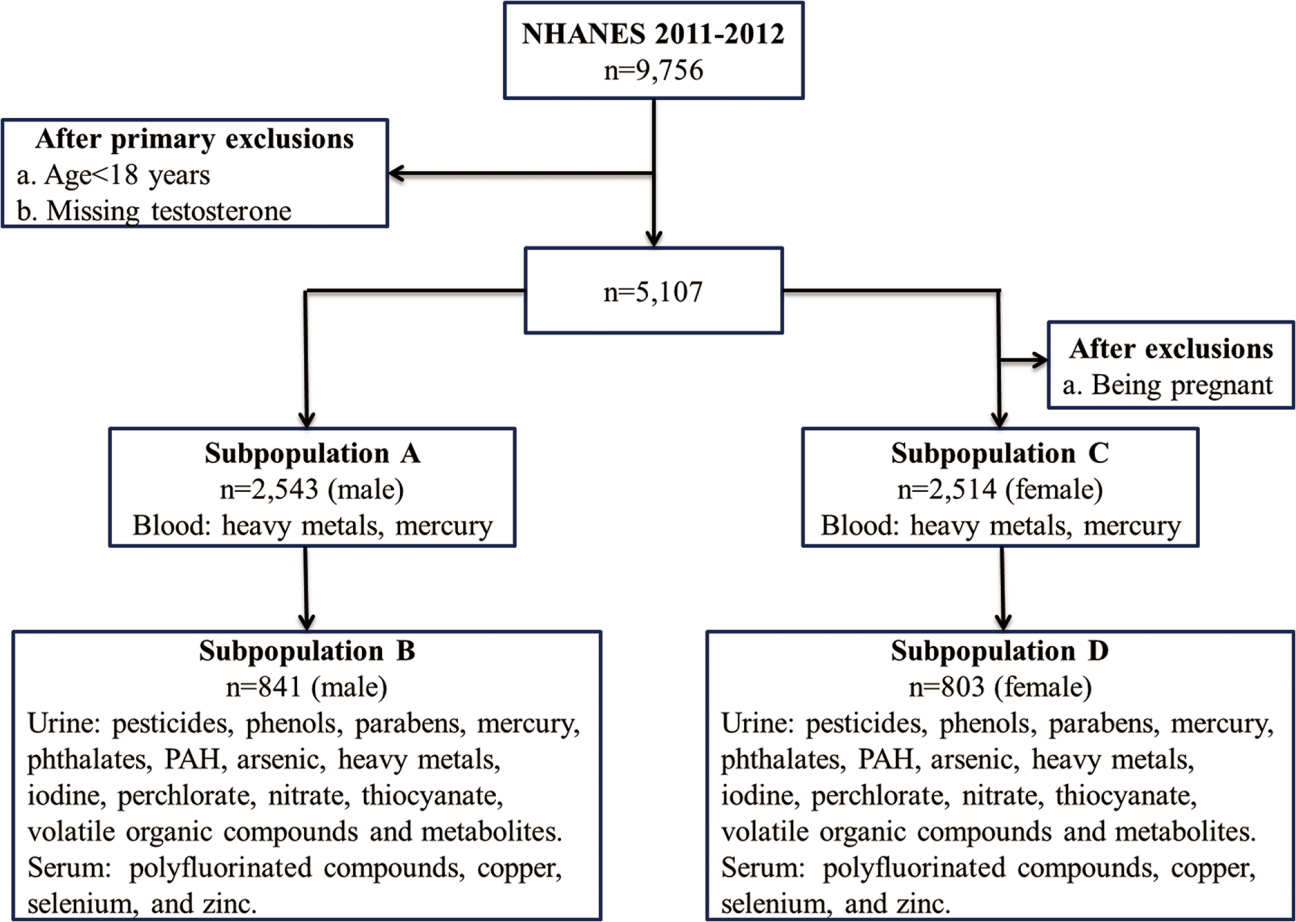
Schematic diagram depicting the number of adult subjects who participated in the NHANES 2011–2012 and environmental chemical subclasses that were analyzed. The number of subjects varied mostly because of missing response variables.

There were two primary exclusion criteria of subjects, including an age less than 18 years and a lack of testosterone measurement. Therefore, the population of interest that was finally studied was 5,107 subjects. This population was further subdivided by sex. Fifty pregnant women were also excluded in the female group. Not all urinary or blood chemicals that were investigated could actually be detected in all of the subjects. The final sample size is summarized in Fig 1. Approval for our analysis of the NHANES data was granted by the University of Louisville Institutional Review Board. The study was approved by the Centers for Disease Control and Prevention’s institutional review board. Study activities were approved by the National Center for Health Statistics (NCHS) institutional review board.

### Chemicals

All of the survey data posted by the NCHS before July 2014 were downloaded. This yielded 110 chemicals in 15 subclasses, as categorized by the NCHS (S1 Table). The chemicals included heavy metals (lead, cadmium, total mercury, selenium, and manganese) and mercury compounds (inorganic, ethyl, and methyl) in blood, pesticides, phenols, parabens, phthalates and plasticizer metabolites, total arsenic and speciated arsenic, polyaromatic hydrocarbons, perchlorate, nitrate, thiocyanate, iodine, volatile organic compounds, metabolites in urine, and polyfluorinated compounds. Copper, selenium, and zinc in serum were also included.

All testosterone and chemical levels were detected on the same day from each participant. We assessed only chemicals with a ≥60% detection rate to avoid bias in calculating chemicals with levels lower than the lower limit of detection [19]. This yielded 82 chemicals of interest from the original 110 chemicals (see S1 Table).

### Covariants

We controlled for the following *a priori* confounders of the relation between environmental chemicals and testosterone levels: age, ethnicity, urinary creatinine, serum cotinine, the poverty income ratio (PIR), body mass index (BMI), and alcohol.

To account for variation in dilution in spot urinary samples, urinary creatinine was adjusted as an independent variable, as suggested by previous studies [20]. Serum cotinine, a marker of exposure to environmental tobacco smoke, was categorized as less than the limit of detection (0.015 ng/mL), low exposure (0.015–10 ng/mL), and high exposure (≥10 ng/mL) [21]. The PIR was calculated by dividing household income by the poverty guidelines specific to the survey year. We also evaluated the PIR as a potential confounder in three categories: low (< 1.3), middle (1.3–3.5) and high (≥3.5). BMI was evaluated in tertiles according to the following categories: normal (<25 kg/m^2^), overweight (25–30 kg/m^2^), and obese (≥30 kg/m^2^). Because alcohol intake is associated with serum testosterone levels [22], it was added as a covariant (gram).

### Outcome variables and statistical methods

From 1999, NHANES became a continuous survey. Approximately 5,000 individuals of all ages are interviewed and complete a health examination component of the survey every year. The NHANES data include 110 environmental chemicals in 15 subclasses in either blood or urine. As in the latest published data of NHANES, serum testosterone levels were first to be added in the survey since 1999. Total serum testosterone levels were detected by the isotope dilution liquid chromatography tandem mass spectrometry method, based on the National Institute for Standards and Technology’s reference method. The distribution of testosterone was skewed, and the Shapiro–Wilk test was below 0.05, even after various transformations. Therefore, testosterone was dichotomized at the median concentration (374.51 ng/dL) into low and high (low = 1, high = 0) levels.

We summed the ratings of all of the chemicals within each of the 15 subclasses to integrate their combined exposure [23]. For each chemical subclass, subjects were classified into quartiles by their combined exposure, with the first quartile representing subjects with the lowest levels, or below the limit of detection. Logistic regression, with the first quartile as the reference to calculate multivariate adjusted odds ratios (ORs) for testosterone concentrations, was performed. ORs were adjusted for age (years), ethnicity (Mexican-American, other Hispanic, non-Hispanic White, non-Hispanic Black, and Other Race—including multi-racial), PIR (tertiles), alcohol (gram), creatinine levels (mg/dL), cotinine (tertiles), and BMI (tertiles). Linear trends in the OR of testosterone levels across increasing urinary environmental chemical quartiles were determined by modeling chemicals as an ordinal variable. Individual environmental chemicals were evaluated further if trend tests for the association between the entire subclass and testosterone concentrations were statistically significant. SAS, version 9.2 (SAS Institute Inc., Cary, NC, USA) was used for all statistical analyses. A *P* value <0.05 was set as the threshold of statistical significance.

## Results

### Demographic information

The mean and standard deviation (continuous variables) and frequency and percentage (categorical variables) of the subjects’ characteristics in adult men from the NHANES 2011–2012 database (n = 2,543) are shown in Table 1.

**Table 1:** Characteristics of 2543 adult men by testosterone in NHANES 2011–2012.

Characteristics	Low testosterone^a^ (n = 1,273)	High testosterone (n = 1,270)	P-value^b^
**Age**	49.88±18.11	44.27±18.66	<0.001
**Urine creatinine**	140.01±79.29	138.18±90.78	0.258
**Alcohol (gram)**	8.46±19.15	14.31±32.53	0.004
**Race**			0.312
**Mexican American**	138 (10.8)	141 (11.1)	
**Other Hispanic**	130 (10.2)	117 (9.2)	
**Non-Hispanic White**	499 (39.2)	460 (36.2)	
**Non-Hispanic Black**	292 (22.9)	330 (26.0)	
**Other Race—Including Multi-Racial**	214 (16.8)	222 (17.5)	
**BMI (kg/m** ^**2**^ **)**	30.37±6.44	26.09±4.69	<0.001
***Category***			<0.001
**<25**	244 (19.2)	572 (45.0)	
**25–30**	452 (35.5)	452 (35.6)	
**≥30**	561 (44.1)	232 (18.3)	
**PIR**	2.53±1.67	2.39±1.66	0.045
***Category***			0.347
**<1.3**	389 (30.6)	422 (33.2)	
**1.3–3.5**	394 (31.0)	393 (30.9)	
**≥3.5**	373 (29.3)	349 (27.5)	
**Serum cotinine (ng/mL)**	53.96±130.28	76.75±138.23	<0.001
***Category***			<0.001
**<0.015**	352 (27.7)	238 (18.7)	
**0.015–10**	613 (48.2)	585 (46.1)	
**≥10**	307 (24.1)	445 (35.0)	

a Values are mean ± SD for continuous and n (%) for categorical variables.

b Chi-square for categorical variables; t-test (for normally distributed) or Wilcoxon–Mann–Whitney (for non-normal distribution) for continuous variables.

According to the median concentration of testosterone (374.51 ng/dL), subjects were divided into low and high levels. As expected, high testosterone levels were more common in younger men than in older men (49.88% vs. 44.27%; *P*<0.001). In the low testosterone group, 10.8% of subjects were Mexican-American, 10.2% were other Hispanic, 39.2% were non-Hispanic White, 22.9% were non-Hispanic Black, and 16.8% were other ethnicities (including multi-racial).

More obese individuals had low testosterone levels (44.1% vs. 18.3%; *P*<0.001). Serum cotinine concentrations were higher in the high testosterone group than in the low testosterone group (35.0% vs. 24.1%; *P*<0.001). There was no difference in the frequency of participants who drank alcohol or families with income at or below the poverty level (PIR <1.3) between the low and high testosterone groups. There was no significant association between chemical exposure and testosterone concentrations in women. Therefore, further analysis was only performed in men in this study.

### Chemical subclasses

Among all of the chemicals that were analyzed, two subclasses, urinary heavy metals and volatile organic compounds and metabolites, showed a suggestive association for the adjusted ORs with testosterone levels (Table 2). The highest quartile of combined heavy metal exposure showed an increased adjusted OR for low testosterone levels of 1.65 compared with the lowest quartile (95% confidence interval [CI]: 0.87, 3.12; *P*_*trend*_ = 0.012). Testosterone was also related to urinary volatile organic compounds and metabolites. The adjusted OR for the highest versus lowest quartiles of exposure was 1.49 (95% CI: 0.73, 3.04; *P*_*trend*_ = 0.039).

**Table 2:** Adjusted ORs (95% CIs) for levels of lower serum testosterone by exposure quartile for pollutant subclasses in adult men.

			Quartile [OR (95% CI)]				
Chemical subclass	n	Chemicals screened/analyzed	First	Second	Third	Fourth	p-value Trend
**Heavy metal (urine)**	836	13/12	Ref	0.98(0.59, 1.63)	0.84(0.49, 1.45)	1.65(0.87, 3.12)	*0*.*012*
**Volatile Organic Compounds and Metabolites(urine)**	833	28/19	Ref	1.28(0.76, 2.15)	1.33(0.74, 2.36)	1.49(0.73, 3.04)	*0*.*039*
**Perchlorate, Nitrate, Thiocyanate(urine)**	833	3/3	Ref	1.40(0.85, 2.31)	1.57(0.89, 2.76)	2.15(1.13, 4.07)	0.245
**Environment pesticides(urine)**	840	2/2	Ref	1.13(0.68, 1.89)	1.12(0.66, 1.90)	1.17(0.68, 2.03)	0.286
**Environment parabens(urine)**	840	4/4	Ref	1.84(1.12, 3.04)	1.52(0.91, 2.54)	1.67(0.97, 2.88)	0.177
**Copper, Selenium, and Zinc (serum)**	832	3/3	Ref	0.91(0.55, 1.50)	0.94(0.57, 1.56)	0.77(0.46, 1.30)	0.712
**Heavy metal (blood)**	2541	5/5	Ref	1.11(0.67, 1.84)	0.58(0.35, 0.96)	0.59(0.35, 0.99)	0.156
**Polyfluorinated compounds (serum)**	824	12/6	Ref	1.01(0.61, 1.67)	0.85(0.51, 1.40)	1.19(0.71, 2.00)	0.494
**Polyaromatic Hydrocarbons(urine)**	833	10/10	Ref	1.70(1.01, 2.86)	1.20(0.70, 2.08)	1.44(0.74, 2.79)	0.961
**Total mercury(urine)**	839	1/1	Ref	0.64(0.38, 1.07)	0.92(0.53, 1.60)	0.72(0.41, 1.27)	0.654
**Environment phenols(urine)**	840	3/3	Ref	0.93(0.56, 1.55)	1.40(0.83, 2.36)	1.01(0.58, 1.74)	0.610
**Phthalatesand Plasticizers Metabolites (urine)**	840	14/12	Ref	0.90(0.54, 1.49)	0.99(0.57, 1.73)	1.25(0.71, 2.22)	0.970
**Iodine(urine)**	839	1/1	Ref	1.64(0.97, 2.75)	1.36(0.79, 2.34)	1.27(0.72, 2.25)	0.886
**Total and speciated arsenics(urine)**	840	8/2	Ref	1.56(0.93, 2.61)	1.33(0.77, 2.28)	1.23(0.67, 2.25)	0.875
**Mercury[inorganic,ethyl, methyl] (blood)**	2539	3/1	Ref	1.06(0.65, 1.72)	0.97(0.59, 1.61)	0.81(0.48, 1.39)	0.499

Ref: referent. ORs were adjusted for age (years), race (Mexican American, Other Hispanic, Non-Hispanic White, Non-Hispanic Black, and Non-Hispanic Black), poverty income ratio (tertiles), alcohol (gram), creatinine (mg/dL), cotinine (tertiles) and BMI (tertiles). Serum and blood chemicals were adjusted above-mentionedsame covariants expect for the creatininevariable.

### Individual chemical

**Fig 2:**
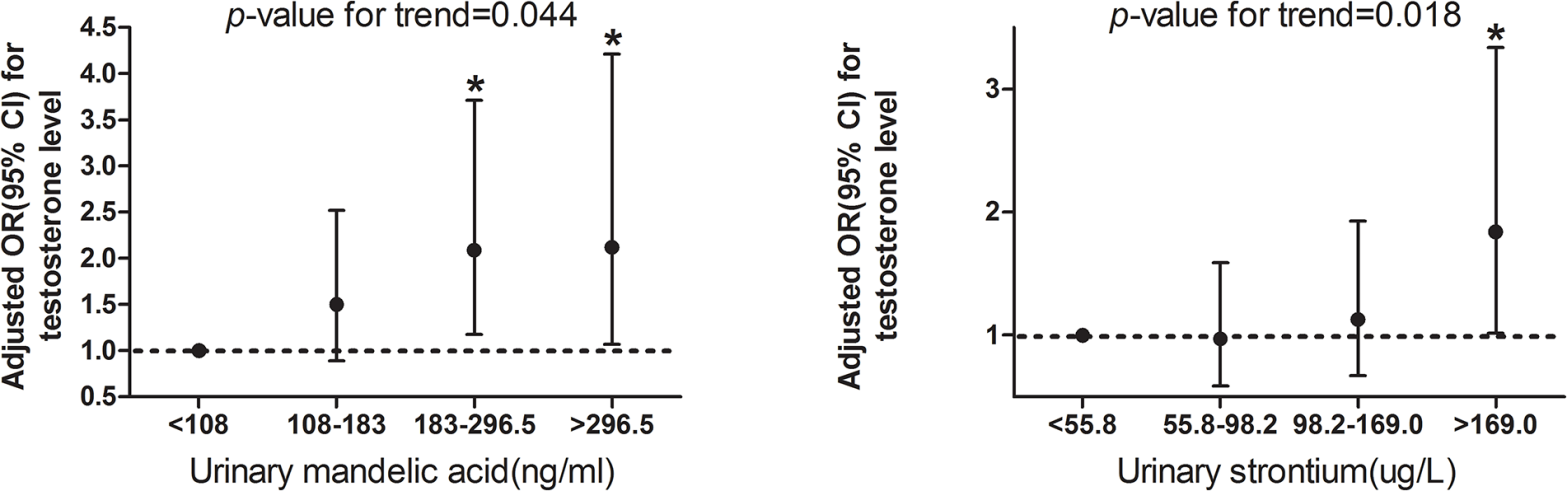
Adjusted ORs and 95% CIs for testosterone levels by increasing quintiles of urinary mandelic acid (A) and strontium (B). *P<0.05.

Fig 2 shows urinary mandelic acid and strontium levels as a function of the OR for low testosterone levels in men. The distribution of mandelic acid, and strontium in this population is shown in S1 Fig. Trend analysis showed an increased risk of having low testosterone levels with increasing urinary mandelic acid levels and strontium levels (Fig 2A and 2B). The detection rates were high for urinary mandelic and strontium (100%). Exposure to the rest of the heavy metals and urinary volatile organic compounds and metabolites was not associated with high testosterone concentrations (S2 Table).

For the observed association between urinary mandelic acid quartiles and low testosterone concentrations in adult men, the adjusted OR for the highest versus lowest quartiles of exposure was 2.12 (95% CI: 1.07, 4.21; *P*_*trend*_ = 0.044). Similary, the OR for strontrium was 1.84 (95% CI: 1.02, 3.34; P_trend_ = 0.018). The remaining 18 compounds and metabolites had no significant associations (S2 Table).

## Discussion

In this study, we examined the association of environmental chemicals and serum testosterone levels based on NHANES data. After comparing the relationship between 110 types of different chemical levels and serum testosterone levels, we found, for the first time, that urinary mandelic acid, and strontium levels were inversely associated with serum testosterone levels.

Mandelic acid, an aromatic alpha hydroxy acid, is considered a biomarker of styrene and related products exposure [24]. Mandelic acid is an intermediate chemical that is currently widely used in the synthetic rubber and plastic industries. Studies have shown that exposure to styrene disrupts sperm DNA integrity in styrene workers and thus affects male reproductive capacity [25, 26]. After *in utero* exposure of styrene trimers in rats, the male pups have increased testosterone levels compared with controls [27]. Conversely, testosterone levels are decreased with intraperitoneal injection of styrene in adult male rats [28]. The urinary geometric mean of mandelic acid is approximately 443 μg/g (creatinine adjusted) in the Italian population [29]. This value is higher than that in the NHANES population that we studied (creatinine adjusted genomic mean: 157 μg/g). However, in workers who have intensive contact with styrene, the level of mandelic acid is extremely high (94.2 mg/g) [30]. There are no relevant epidemiological studies on the relationship between mandelic acid and testosterone levels in the general population. We showed, for the first time, that elevated urinary mandelic acid levels are associated with low testosterone levels in men, even at a low dose of 157 ug/g, using the NHANES data. This result may help extend insight into the biological effects of styrene.

Strontium is a non-radioactive silvery metal that is naturally found in the human body as a trace element. Approximately 99% of strontium in the human body is sequestered in the bones. In daily life, strontium chloride hexahydrate acts as an analgesic, and is added to toothpaste. Several different forms of strontium are used in medicine, specifically for treatment of prostate and bone cancer, where patients are administered intravenous radioactive strontium-89. Strontium causes side effects in kidney disease [31]. However, no toxic symptoms caused by dietary strontium have been reported in animal studies [32]. Little information regarding the safety of strontium chloride on reproductive or developmental effects in humans is available. The previous study of investigating urine metabolite among normal population indicated the strontium concentration was 0.163 (0.030–0.415) (mean and range) umol/mmol creatinine, while our data shown the strontium level was 0.138 (0.130–0.145). It was similar with other research [33]. Our study showed that increased urinary strontium levels were significantly associated with low testosterone levels.

Zeng et al [16] found that urinary manganese was associated with decreased testosterone level. Less sample size and the different ethnicities between studies might be the reasons leading to inconsistency of results compared to our present study.

Androgen deficiency, manifesting as low testosterone levels, has side effects on human health, with specific signs and symptoms, such as osteoporosis, oligospermia or azoospermia, and gynecomastia. There are no reports on the association between urinary mandelic acid, and strontium with testosterone levels in humans. Because of the cross-sectional nature of the present study, we could not assess whether mandelic acid, and strontium affect testosterone levels or vice versa. Our study suggests a relationship between environmental chemical exposure and low testosterone levels. The findings from the present study highlight the need for additional research to evaluate the association between long-term exposure to physiological levels of urinary mandelic acid, and strontium, and low testosterone levels in adult men in the USA. Future studies should be performed to confirm the potential role of these environmental chemicals in reduced testosterone levels.

## Supporting Information

S1 Fig Distribution of urinary creatinine-adjusted mandelic acid (A), and strontium (B) levels in adult men. The left panel shows chemicals without logarithmic transformation. The right panel shows chemicals with logarithmic transformation.
(TIF)

S1 Table Distribution of serum or urinary environmental chemicals concentrations in male adults. (DOC)

S2 Table Adjusted Odds Ratios (ORs) and 95% CIs for levels of serum testosterone by exposure quartiles for urinary volatile organic compounds and metabolites and heavy metal in adult males NHANES 2011–2012. ORs were adjusted for age(years), race (Mexican American, Other Hispanic, Non-Hispanic White, Non-Hispanic Black, and Non-Hispanic Black), poverty income ratio (tertiles), alcohol (gram), creatinine(mg/dL), cotinine (tertiles) and BMI (tertiles). *Statistically significant (p < 0.05).
(DOC)
