# Supplementary figures and images for: Low Serum Testosterone Levels Are Associated with Elevated Urinary Mandelic Acid, and Strontium Levels in Adult Men According to the US 2011–2012 National Health and Nutrition Examination Survey
Source: PLoS One. 2015 May 21;10(5):e0127451. doi: 10.1371/journal.pone.0127451 (PMC4440739; doi:10.1371/journal.pone.0127451)

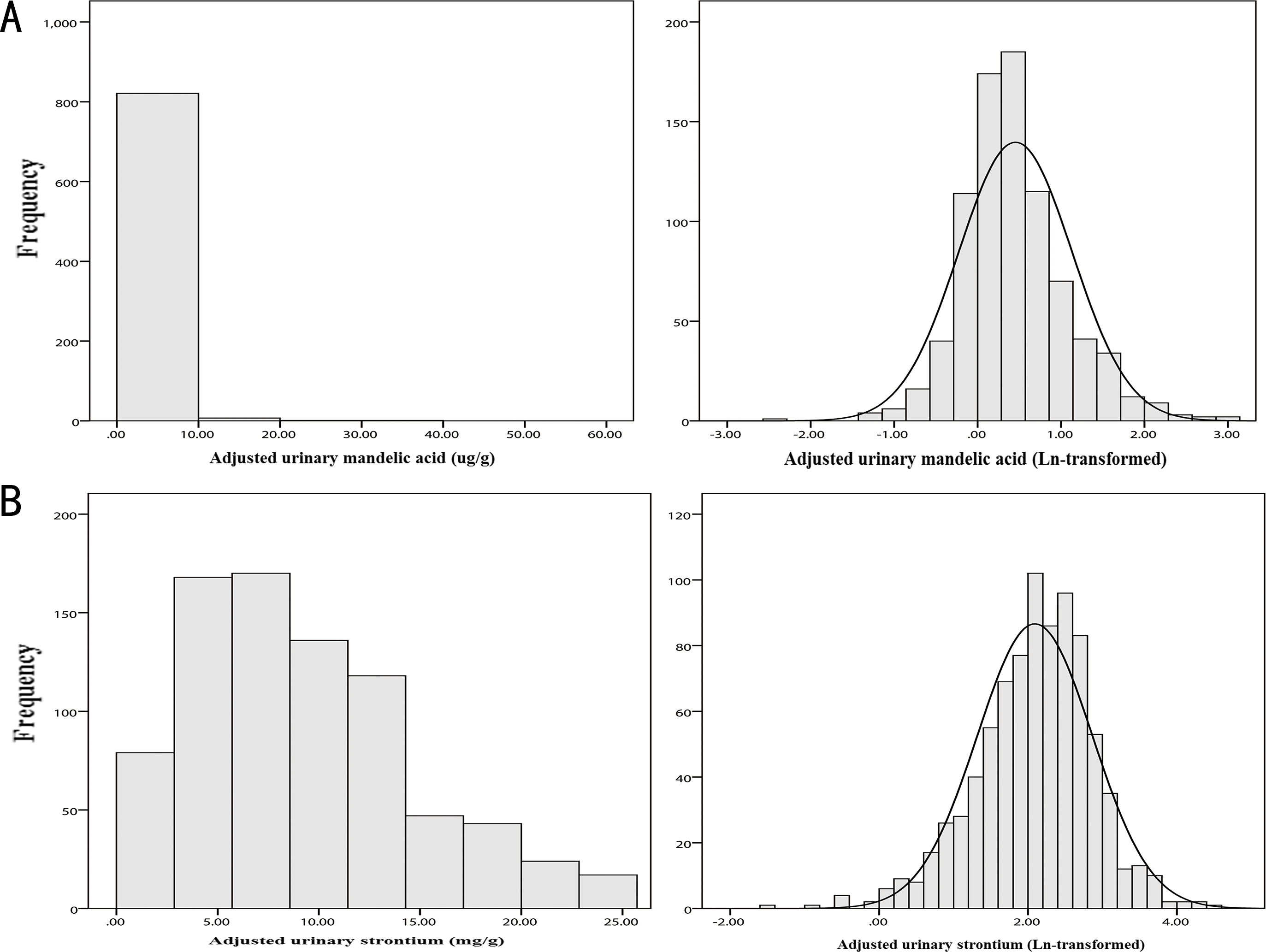

Supplement: S1 Fig — The left panel shows chemicals without logarithmic transformation. The right panel shows chemicals with logarithmic transformation. (TIF) [file pone.0127451.s001.tif]
