# Supplementary material for: Low Serum Testosterone Levels Are Associated with Elevated Urinary Mandelic Acid, and Strontium Levels in Adult Men According to the US 2011–2012 National Health and Nutrition Examination Survey
Source: PLoS One. 2015 May 21;10(5):e0127451. doi: 10.1371/journal.pone.0127451 (PMC4440739; doi:10.1371/journal.pone.0127451)
Supplement: S1 Table — (DOC) [file pone.0127451.s002.doc]

S1 Table. Distribution of serum or urinary environmental chemicals concentrations in male adults.

| Analyte | Percent >LOD | Concentration[mean (range)] |
| --- | --- | --- |
| **Blood heavy metal** |  |  |
| lead (ug/dL) | 99.96 | 1.78(0.18-61.29) |
| cadmium (ug/L) | 99.92 | 0.52(0.11-9.30) |
| mercury, total (ug/L) | 99.92 | 1.79(0.11-50.81) |
| selenium (ug/L) | 100 | 195.61(105.77-635.84) |
| manganese (ug/L) | 100 | 9.12(1.61-45.49) |
| **Blood mercury** |  |  |
| Mercury, inorganic (ug/L) | 24.62 | 0.27(0.19-4.10) |
| Mercury, ethyl (ug/L) | 3.51 | 0.12(0.11-0.95) |
| Mercury, methyl (ug/L) | 88.81 | 1.68(0.08-52.92) |
| **Urinary pesticides** |  |  |
| 2,5-dichlorophenol (ug/L) | 95.60 | 119.90(0.14-25300.00) |
| 2,4-dichlorophenol (ug/L) | 82.62 | 3.82(0.14-562.00) |
| **Urinary phenols** |  |  |
| Benzophenone-3 (ng/mL) | 96.43 | 168.21(0.28-32500.00) |
| Bisphenol A (ng/mL) | 87.98 | 3.20(0.28-105.00) |
| Triclosan (ng/mL) | 71.55 | 89.49(1.63-3570.00) |
| **Urinary parabens** |  |  |
| Butyl paraben (ng/ml) | 11.67 | 1.24(0.14-308.00) |
| Ethyl paraben (ng/ml) | 36.07 | 9.59(0.71-844.00) |
| Methyl paraben (ng/ml) | 98.93 | 173.10(0.71-7860.00) |
| Propyl paraben (ng/ml) | 87.74 | 39.45(0.14-2030.00) |
| **Serum Polyfluorinated Compounds** |  |  |
| 2-(N-ethyl-PFOSA) acetate(ng/mL) | 0.05 | 0.077(0.070-0.510) |
| Perfluorodecanoic acid(ng/mL) | 86.67 | 0.36(0.07-17.08) |
| Perfluorooctanoic acid(ng/mL) | 99.16 | 2.81(0.07-43.00) |
| Perfluorooctane sulfonic acid(ng/mL) | 99.52 | 11.69(0.14-235.00) |
| Perfluorohexane sulfonic acid(ng/mL) | 98.44 | 2.31(0.07-47.80) |
| 2-(N-methyl-PFOSA) acetate(ng/mL) | 53.42 | 0.24(0.06-4.12) |
| Perfluorobutane sulfonic acid(ng/mL) | 0.96 | 0.07(0.07-0.43) |
| Perfluoroheptanoic acid(ng/mL) | 15.25 | 0.09(0.07-1.34) |
| Perfluorononanoic acid(ng/mL) | 99.04 | 1.25(0.06-10.50) |
| Perfluorooctane sulfonamide(ng/mL) | 1.08 | 0.07(0.07-0.62) |
| Perfluoroundecanoic acid(ng/mL) | 61.10 | 0.28(0.07-6.91) |
| Perfluorododecanoic acid(ng/mL) | 9.00 | 0.09(0.07-1.35) |
| **Urinary mercury** |  |  |
| Mercury (ug/L) | 95.24 | 0.59(0.04-7.54) |
| **Urinary heavy metal** |  |  |
| Barium, urine (ug/L) | 98.81 | 1.83(0.07-87.40) |
| Cadmium, urine (ug/L) | 98.10 | 0.34(0.04-4.83) |
| Cobalt, urine (ug/L) | 99.17 | 0.37(0.03-5.97) |
| Cesium, urine (ug/L) | 100 | 5.09(0.21-29.70) |
| Molybdenum, urine (ug/L) | 100 | 57.43(4.34-541.00) |
| Manganese, urine (ug/L) | 65.76 | 0.15(0.06-4.28) |
| Lead, urine (ug/L) | 96.67 | 0.74(0.06-35.00) |
| Antimony, urine (ug/L) | 58.50 | 0.08(0.03-2.34) |
| Tin, urine (ug/L) | 83.23 | 1.38(0.16-55.01) |
| Strontium, urine (ug/L) | 100 | 123.58(4.74-615.90) |
| Thallium, urine (ug/L) | 99.52 | 0.20(0.01-1.29) |
| Tungsten, urine (ug/L) | 86.33 | 0.13(0.02-1.68) |
| Uranium, urinary (ug/L) | 74.67 | 0.0119(0.0023-0.7222) |
| **Urinary Phthalates and Plasticizers Metabolites** |  |  |
| Mono(carboxynonyl) Phthalate (ng/mL) | 98.57 | 4.9(0.14-132.90) |
| Mono(carboxyoctyl) Phthalate (ng/mL) | 100 | 56.96(0.60-1773.50) |
| Mono-2-ethyl-5-carboxypentyl phthalate(ng/mL) | 99.64 | 27.06(0.14-1548.40) |
| Mono-n-butyl phthalate (ng/mL) | 93.69 | 22.59(0.28-2462.30) |
| Mono-(3-carboxypropyl) phthalate(ng/mL) | 98.33 | 17.05(0.14-1537.90) |
| Mono-ethyl phthalate (ng/mL) | 100 | 192.88(1.10-10074.00) |
| Mono-(2-ethyl-5-hydroxyhexyl) phthalate(ng/mL) | 99.52 | 19.39(0.14-1186.60) |
| Mono-(2-ethyl)-hexyl phthalate (ng/mL) | 78.33 | 3.50(0.35-125.20) |
| Mono-n-methyl phthalate(ng/mL) | 61.90 | 3.37(0.35-185.20) |
| Mono-isononyl phthalate (ng/mL) | 55.83 | 4.91(0.35-305.80) |
| Mono-(2-ethyl-5-oxohexyl) phthalate(ng/mL) | 99.52 | 11.22(0.14-528.70) |
| Mono-benzyl phthalate (ng/mL) | 96.67 | 9.49(0.21-225.20) |
| Mono-isobutyl phthalate(ng/mL) | 98.69 | 12.52(0.14-196.50) |
| Cyclohexane-1,2-dicarboxylic acid monohydroxyisononyl ester(MHNC) (ng/mL) | 16.07 | 0.48(0.28-39.00) |
| **Urinary Total Arsenic and Speciated Arsenics** |  |  |
| Urinary arsenic, total (ug/L) | 97.38 | 23.01(0.88-1035.80) |
| Urinary Arsenous acid (ug/L) | 39.24 | 0.66(0.34-66.30) |
| Urinary Arsenic acid (ug/L) | 3.80 | 0.70(0.62-42.50) |
| Urinary Arsenobetaine (ug/L) | 51.61 | 13.77(0.84-930.00) |
| Urinary Arsenocholine (ug/L) | 5.11 | 0.28(0.20-11.30) |
| Urinary Dimethylarsonic acid (ug/L) | 83.23 | 6.71(1.27-136.00) |
| Urinary Monomethylacrsonic acid (ug/L) | 32.82 | 1.04(0.63-72.90) |
| Urinary Trimethylarsine Oxide (ug/L) | 1.55 | 0.19(0.18-6.34) |
| **Urinary Polyaromatic Hydrocarbons** |  |  |
| 1-hydroxynaphthalene (ng/L) | 100 | 9213.54(81.00-1749766) |
| 2-hydroxynaphthalene (ng/L) | 100 | 8882.08(183-115584) |
| 3-hydroxyfluorene (ng/L) | 99.16 | 349.74(7.10-6541.00) |
| 2-hydroxyfluorene (ng/L) | 100 | 676.64(19.00-15068.00) |
| 3-hydroxyphenanthrene (ng/L) | 98.09 | 146.35(7.10-6683.00) |
| 1-hydroxyphenanthrene (ng/L) | 100 | 214.38(12.00-7475.00) |
| 2-hydroxyphenanthrene (ng/L) | 99.16 | 124.35(7.10-3262.00) |
| 1-hydroxypyrene (ng/L) | 98.81 | 220.60(7.10-7574.00) |
| 9-hydroxyfluorene (ng/L) | 100 | 572.19(22.00-16401.00) |
| 4-phenanthrene (ng/L) | 79.90 | 36.42(7.10-705.00) |
| **Urinary Perchlorate, Nitrate, Thiocyanate** |  |  |
| Urinary perchlorate (ng/mL) | 100 | 5.23(0.14-104.00) |
| Urinary nitrate (ng/mL) | 100 | 60455.89(3170-1050000) |
| Urinary thiocyanate (ng/mL) | 100 | 2290.95(35.00-46600.00) |
| **Serum Copper, Selenium, and Zinc** |  |  |
| Serum Copper (ug/dL) | 100 | 105.56(44.40-258.20) |
| Serum Selenium (ug/L) | 100 | 130.69(70.60-299.10) |
| Serum Zinc (ug/dL) | 100 | 83.92(47.40-232.50) |
| **Urinary Iodine** |  |  |
| Iodine, urine (ug/L) | 100 | 235.30(12.60-9321.90) |
| **Urinary Volatile Organic Compounds and Metabolites** |  |  |
| N-acel-S-(1,2-dichlorovinl)-L-cys(ng/mL) | 0.12 | 8.91(8.91-14.30) |
| N-Acel-S-(2,2-Dichlorvinyl)-L-cys(ng/mL) | 3.60 | 4.64(4.60-26.80) |
| 2-Methylhippuric acid (ng/mL) | 97.60 | 96.73(3.54-18200.00) |
| 3-methipurcacd& 4-methipurcacd(ng/mL) | 100 | 595.33(10.30-55000.00) |
| N-Ace-S-(2-carbamoylethyl)-L-cys(ng/mL) | 100 | 97.78(2.41-3120.00) |
| N-Ace-S-(N-methlcarbamoyl)-L-cys(ng/mL) | 99.89 | 235.81(3.89-2230.00) |
| 2-amnothiazolne-4-carbxylic acid(ng/mL) | 93.64 | 137.34(10.60-1250.00) |
| N-Acetyl-S-(benzyl)-L-cysteine(ng/mL) | 99.40 | 13.37(0.35-649.00) |
| N-Acetyl-S-(n-propyl)-L-cysteine(ng/mL) | 81.40 | 15.08(0.85-545.00) |
| N-Acetyl-S-(2-Carbxyethyl)-L-Cys(ng/mL) | 99.76 | 204.46(5.66-3390.00) |
| N-acetyl-S-(2-cyanoethyl)-L-cyst(ng/mL) | 94.72 | 58.07(0.35-1370.00) |
| N-Ace-S- (3,4-Dihidxybutl)-L-Cys(ng/mL) | 100 | 384.28(14.90-1940.00) |
| N-Ace-S-(dimethylphenyl)-L-Cys(ng/mL) | 3.60 | 0.36(0.35-2.13) |
| N-ac-S-(2-carbmo-2-hydxel)-L-cys(ng/mL) | 72.75 | 23.99(6.65-787.00) |
| N-Ace-S-(2-Hydroxyethyl)-L-cys(ng/mL) | 55.22 | 1.28(0.42-17.00) |
| N-Ace-S-(2-hydroxypropyl)-L-cys(ng/mL) | 99.89 | 156.30(0.92-17100.00) |
| N-Ace-S-(3-Hydroxypropyl)-L-Cys(ng/mL) | 100 | 706.19(20.70-8320.00) |
| N-A-S-(3-hydrxprpl-1-metl)-L-cys(ng/mL) | 100 | 1027.58(23.70-15200.00) |
| Mandelic acid(ng/mL) | 99.28 | 320.00(8.50-31900.00) |
| N-A-S-(4-hydrxy-2butn-l-yl)-L-cys(ng/mL) | 99.16 | 29.74(0.42-423.00) |
| N-Ac-S-(2-Hydrxy-3-butnyl)-L-Cys(ng/mL) | 14.65 | 0.91(0.50-22.90) |
| N-A-S-(1-HydrxMet)-2-Prpn)-L-Cys(ng/mL) | 1.08 | 0.50(0.50-2.22) |
| t,t-Muconic acid(ng/mL) | 90.76 | 185.90(14.10-8020.00) |
| N-ace-S-(phenl-2-hydxyetl)-L-cys(ng/mL) | 37.33 | 1.99(0.50-550.00) |
| Phenylglyoxylic acid(ng/mL) | 98.92 | 356.12(8.49-32200.00) |
| N-Acetyl-S-(phenyl)-L-cysteine(ng/mL) | 51.74 | 0.98(0.42-15.30) |
| N-Acetyl-S-(trichlorovinyl)-L-cys(ng/mL) | 0.12 | 2.12(2.12-4.74) |
| 2-thoxothazlidne-4-carbxylic acid(ng/mL) | 77.43 | 35.26(2.47-966.00) |
