# Supplementary material for: Low Serum Testosterone Levels Are Associated with Elevated Urinary Mandelic Acid, and Strontium Levels in Adult Men According to the US 2011–2012 National Health and Nutrition Examination Survey
Source: PLoS One. 2015 May 21;10(5):e0127451. doi: 10.1371/journal.pone.0127451 (PMC4440739; doi:10.1371/journal.pone.0127451)
Supplement: S2 Table — ORs were adjusted for age(years), race (Mexican American, Other Hispanic, Non-Hispanic White, Non-Hispanic Black, and Non-Hispanic Black), poverty income ratio (tertiles), alcohol (gram), creatinine(mg/dL), cotinine (tertiles) and BMI (tertiles). *Statistically significant (p < 0.05). (DOC) [file pone.0127451.s003.doc]

S2 Table. Adjusted Odds Ratios (ORs) and 95% CIs for levels of serum testosterone by exposure quartiles for urinary volatile organic compounds and metabolites and heavy metal in adult males NHANES 2011-2012.

|  |  |  | Quartile [ OR (95% CI)] | | | |  |
| --- | --- | --- | --- | --- | --- | --- | --- |
| Chemical subclass | n | Detection rates | First | Second | Third | Fourth | p-value Trend |
| **Volatile Organic Compounds and Metabolites (urine)(ng/mL)** |  |  |  |  |  |  |  |
| Urinary 2-Methylhippuric acid | 833 | 97.60% | Referent | 0.98(0.59, 1.63) | 0.89(0.53, 1.48) | 1.22(0.69, 2.17) | *0.094* |
| Urinary 3-methipurcacd& 4-methipurc acid | 833 | 100% | Referent | 0.92(0.55, 1.54) | 0.84(0.49, 1.44) | 0.94(0.52, 1.70) | *0.250* |
| Urinary N-Ace-S-(2-carbamoylethyl)-L-cys | 833 | 100% | Referent | 0.79(0.48, 1.32) | 1.03(0.60, 1.78) | 1.02(0.55, 1.91) | *0.337* |
| Urinary N-Ace-S-(N-methlcarbamoyl)-L-cys | 833 | 99.89% | Referent | 0.93(0.55, 1.58) | 1.24(0.71, 2.14) | 1.40(0.72, 2.74) | *0.129* |
| Urinary 2-amnothiazolne-4-carbxylic acid | 833 | 93.64% | Referent | 0.90(0.55, 1.48) | 0.92(0.55, 1.54) | 1.13(0.67, 1.92) | *0.301* |
| Urinary N-Acetyl-S-(benzyl)-L-cysteine | 833 | 99.40% | Referent | 1.09(0.65, 1.82) | 0.90(0.51, 1.59) | 1.52(0.82, 2.81) | *0.884* |
| Urinary N-Acetyl-S-(n-propyl)-L-cysteine | 812 | 81.40% | Referent | 0.95(0.58, 1.55) | 1.12(0.67, 1.87) | 0.99(0.58, 1.69) | 0.733 |
| Urinary N-Acetyl-S-(2-Carbxyethyl)-L-Cys | 833 | 99.76% | Referent | 0.85(0.51, 1.43) | 1.24(0.72, 2.17) | 1.00(0.52, 1.91) | *0.190* |
| Urinary N-acetyl-S-(2-cyanoethyl)-L-cyst | 833 | 94.72% | Referent | 1.07(0.64, 1.79) | 1.28(0.73, 2.23) | 0.42(0.17, 1.02) | *0.433* |
| Urinary N-Ace-S- (3,4-Dihidxybutl)-L-Cys | 833 | 100% | Referent | 1.24(0.74, 2.09) | 1.46(0.81, 2.66) | 2.43(1.11, 5.33)* | *0.463* |
| Urinary N-ac-S-(2-carbmo-2-hydxel)-L-cys | 833 | 72.75% | Referent | 0.92(0.55, 1.55) | 1.60(0.93, 2.75) | 1.21(0.65, 2.25) | *0.328* |
| Urinary N-Ace-S-(2-hydroxypropyl)-L-cys | 833 | 99.89% | Referent | 0.93(0.55, 1.55) | 0.86(0.50, 1.48) | 1.14(0.64, 2.05) | *0.722* |
| Urinary N-Ace-S-(3-Hydroxypropyl)-L-Cys | 833 | 100% | Referent | 1.08(0.65, 1.81) | 0.89(0.52, 1.55) | 1.03(0.55, 1.92) | *0.219* |
| Urinary N-A-S-(3-hydrxprpl-1-metl)-L-cys | 833 | 100% | Referent | 0.96(0.58, 1.60) | 1.24(0.69, 2.24) | 1.26(0.66, 2.42) | *0.067* |
| Urinary mandelic acid | 833 | 99.28% | Referent | 1.50(0.89, 2.52) | 2.09(1.18, 3.71)* | 2.12(1.07, 4.21)* | *0.044* |
| Urinary N-A-S-(4-hydrxy-2butn-l-yl)-L-cys | 833 | 99.16% | Referent | 0.91(0.54, 1.52) | 1.64(0.94, 2.85) | 1.12(0.57, 2.21) | *0.365* |
| Urinaryt,t-Muconic acid | 833 | 90.76% | Referent | 1.06(0.64, 1.76) | 1.43(0.83, 2.47) | 2.14(1.20, 3.82)* | *0.243* |
| Urinary phenylglyoxylic acid | 833 | 98.92% | Referent | 1.38(0.81, 2.33) | 2.57(1.41, 4.66)* | 3.20(1.54, 6.66)* | *0.065* |
| Urinary 2-thoxothazlidne-4-carbxylic acid | 833 | 77.43% | Referent | 1.27(0.77, 2.08) | 1.28(0.76, 2.17) | 1.29(0.76, 2.18) | *0.453* |
| **Heavy Metal (urine)(ug/L)** |  |  |  |  |  |  |  |
| Urinary barium | 841 | 98.81% | Referent | 1.44(0.88, 2.38) | 1.21(0.72, 2.02) | 2.10(1.23, 3.60)* | *0.066* |
| Urinary cadmium | 841 | 98.10% | Referent | 1.20(0.71, 2.05) | 1.58(0.90, 2.79) | 1.47(0.74, 2.90) | *0.927* |
| Urinary cobalt | 841 | 99.17% | Referent | 1.36(0.82, 2.27) | 1.34(0.77, 2.35) | 1.63(0.88, 3.00) | *0.514* |
| Urinary cesium | 841 | 100% | Referent | 1.38(0.81, 2.33) | 1.08(0.61, 1.92) | 1.41(0.74, 2.68) | *0.604* |
| Urinary molybdenum | 841 | 100% | Referent | 1.01(0.61, 1.66) | 1.28(0.74, 2.21) | 0.96(0.53, 1.75) | *0.259* |
| Urinary manganese | 841 | 65.76% | Referent | 0.73(0.44, 1.21) | 1.26(0.77, 2.06) | 1.05(0.65, 1.70) | *0.228* |
| Urinary lead | 841 | 96.67% | Referent | 1.01(0.61, 1.68) | 0.64(0.36, 1.11) | 1.08(0.57, 2.04) | *0.763* |
| Urinary tin | 841 | 83.23% | Referent | 1.07(0.64, 1.78) | 1.01(0.59, 1.81) | 1.03(0.59, 1.81) | *0.870* |
| Urinary strontium | 841 | 100% | Referent | 0.97(0.59, 1.59) | 1.13(0.67, 1.93) | 1.84(1.02, 3.34)* | *0.018* |
| Urinary thallium | 841 | 99.52% | Referent | 1.09(0.67, 1.79) | 1.50(0.86, 2.63) | 1.27(0.69, 2.31) | *0.305* |
| Urinary tungsten | 836 | 86.33% | Referent | 1.34(0.81, 2.21) | 1.27(0.75, 2.16) | 1.46(0.81, 2.61) | *0.038* |
| Urinary uranium | 841 | 74.67% | Referent | 0.99(0.60, 1.64) | 1.03(0.61, 1.73) | 1.39(0.78, 2.46) | *0.072* |

ORs were adjusted for age(years), race (Mexican American, Other Hispanic, Non-Hispanic White, Non-Hispanic Black, and Non-Hispanic Black), poverty income ratio (tertiles), alcohol (gram), creatinine(mg/dL), cotinine (tertiles) and BMI (tertiles). *Statistically significant (p < 0.05).
